# Hybrid RSM–NSGA-II based multi-objective optimization of electrical discharge machining of AISI D2 steel

**DOI:** 10.1371/journal.pone.0350415

**Published:** 2026-07-02

**Authors:** Suman Mondal, Biswajit Sing Sardar, Prosun Mandal, S. P. Samal, Biswanath Doloi, Ranjan Kumar Ghadai

**Affiliations:** 1 Mechanical Engineering Department, Ramkrishna Mahato Government Engineering College, Purulia, West Bengal, India; 2 Department of Mechanical Engineering, National Institute of Technology Silchar, Silchar, Assam, India; 3 Department of Biosciences, Saveetha School of Engineering, Saveetha Institute of Medical and Technical Sciences, Chennai, Tamil Nadu, India; 4 Department of Production Engineering, Jadavpur University, Kolkata, West Bengal, India; 5 Manipal Institute of Technology, Manipal Academy of Higher Education, Manipal, Karnataka, India; Ramaiah Institute of Technology, INDIA

## Abstract

In this study, electrical discharge machining (EDM) of AISI D2 die steel was performed by varying three different process parameters: peak current (Ip), pulse-on time (Ton), and duty cycle (c). Enhancing both surface quality and machining performance is very important for die steel applications; therefore, a hybrid approach for multi-objective optimization was employed. A Box–Behnken design of response surface methodology (RSM) was utilized to conduct the experiments, while analysis of variance (ANOVA) was used to examine the influence of process parameters on the responses. Mathematical models were developed using RSM, which were finally utilized as fitness functions for the Non-Dominated Sorting Genetic Algorithm II (NSGA-II) to get solutions of multi-objective optimization. The algorithm generated a set of non-dominated solutions forming the Pareto frontier. To identify the most desirable solution, the Technique for Order Preference by Similarity to Ideal Solution (TOPSIS) was used. The optimal results obtained through TOPSIS analysis were a surface roughness of 5.22 µm and a material removal rate (MRR) of 0.250 g/min, corresponding to the process parameters: peak current (Ip) = 10.03 A, pulse-on time (Ton) = 30.70 µs, and duty cycle (c) = 14.94%.

## Introduction

AISI D2 steel is a high-carbon, high-chromium tool steel that exhibits a good mechanical property such as high wear resistance, superior hardness, good compressive strength, and outstanding dimensional stability. Owing to these characteristics, it is extensively used in the tool and die industry for manufacturing gauges, wear inserts, mould components and other high precision tools. The high chromium content (11–13%) and carbon content (1.4–1.6%) in D2 steel increases its hardenability and wear resistance [1,2]. However, due to its high hardness makes extremely difficult to machine using conventional methods.

Electrical Discharge Machining (EDM), an advanced non-traditional machining technique, is highly suitable for machining such hard-to-cut materials. EDM is widely employed to produce complex shapes with better surface finish by utilizing spark energy, where both the tool and the workpiece must be electrically conductive [3]. The existing literature shows that EDM process parameters—particularly pulse-on time, discharge current, and pulse-off time—having a dominant role in controlling machining performance of AISI D2 steel. Prior studies have primarily focused on identifying influential parameters and analysing their individual effects on responses such as MRR, SR, and TWR. For instance, Kumar et al. [4] and Majhi et al. [5] emphasized the strong influence of pulse-related parameters on performance metrics, while Bachchhav and Gadakh [6] explained the benefits of suitable electrode materials in improving MRR and reducing TWR.

Number of optimization techniques have been applied to enhance EDM performance by identifying appropriate machining parameter combinations. Taguchi-based Grey Relational Analysis (GRA) has been widely adopted for multi-response optimization of EDM processes. For example, multi-response optimization of MRR, SR, and electrode wear rate (EWR) during machining of AISI P20 steel revealed that discharge current was the most influential parameter [7,8]. Similarly, Kumar et al. [9] demonstrated that GRA-based optimization in cryogenically cooled EDM reduced tool wear rate (TWR) by approximately 16% and improved surface roughness by about 19%, with pulse-on time and current identified as dominant control factors. Although effective, these GRA-based approaches are inherently limited by discrete experimental levels and weighted aggregation of responses, which restrict their ability to explore the continuous design space and solve complex optimization problem among conflicting objectives.

To overcome these limitations, hybrid optimization approaches integrating Response Surface Methodology (RSM) with evolutionary algorithms have been explored. Naik et al. [10] combined RSM with a Genetic Algorithm (GA) for optimizing EDM parameters during machining of Al–SiC composites, reporting discharge current as the most significant factor affecting both MRR and SR. However, GA-based optimization typically a single optimal solution, which may not adequately represent the range of requirements in practical multi-objective EDM applications.

Recent investigations have incorporated advanced modelling and prediction techniques to improve EDM performance. Kaigude et al. [11] studied powder-mixed EDM of AISI D2 steel and identified discharge current and pulse-on time as the most significant parameters influencing surface roughness. Machine learning techniques including linear regression, decision trees, and random forests were further employed to predict SR. Abbas et al. [12] also reported that discharge current exerts a stronger influence on surface quality than pulse-on and pulse-off times during EDM of D2 steel using copper and brass electrodes. Deep learning approaches have likewise been explored for surface quality prediction in EDM processes [13]. Despite their strong predictive capabilities, machine learning and deep learning models sometimes show lack transparency and are rarely integrated with systematic multi-objective optimization frameworks.

Conventional statistical tools such as Analysis of Variance (ANOVA) have been extensively used to develop predictive models for MRR, TWR, and SR, consistently identifying discharge current as the most significant EDM parameter across different materials and machining conditions [14]. However, ANOVA-based models primarily focus on parameter significance rather than identifying optimal parameter combinations under multiple conflicting objectives.

Multi-Criteria Decision-Making (MCDM) techniques, including TOPSIS and Distance Analysis (DA), have been applied to EDM optimization to rank alternative parameter settings. These studies reported that TOPSIS is more effective for surface roughness optimization, whereas DA performs better for maximizing MRR. Similarly, CRITIC-based MCDM methods have been employed to optimize EDM turning parameters such as gap current, pulse-on time, rotational speed, and magnetic field assistance for EN24 steel alloys [15–17]. Nevertheless, MCDM approaches rely heavily on predefined weights and limited experimental datasets, which restrict their robustness and generalizability for global optimization.

More advanced Pareto-based evolutionary algorithms have recently been explored for EDM optimization. Khullar et al. [18] and Dikshit et al. [19] successfully employed hybrid RSM–NSGA-II approaches for multi-performance optimization of EDM parameters while machining AISI 5160 steel and Ti–6Al–4V alloy, respectively, demonstrating the effectiveness of NSGA-II in handling conflicting objectives. Mandal et al. [20] applied the Multi-Objective Dragonfly Algorithm (MODA) followed by TOPSIS for EDM optimization of Monel K500, further highlighting the potential of metaheuristic algorithms for multi-objective EDM optimization.

Despite the advancements, the application of a hybrid RSM–NSGA-II framework specifically for EDM of AISI D2 steel remains limited. Given the industrial relevance of AISI D2 steel and the conflicting nature of EDM performance measures, there exists a clear research gap in developing a robust, statistically validated, and Pareto-based multi-objective optimization approach. The present study addresses this gap by integrating RSM-based predictive modelling with NSGA-II to simultaneously optimize MRR, and SR, thereby providing a comprehensive and practical optimization framework for EDM of AISI D2 steel.

## Materials and methods

### Experimentation

In this study, AISI D2 steel was selected as the work material due to its high hardness and wear resistance. A total of 27 specimens were prepared in square plate form with dimensions of 50 mm × 50 mm × 5 mm. The chemical composition of AISI D2 steel is presented in Table 1, and its physical and mechanical properties are listed in Table 2.

**Table 1:** Chemical composition of AISI D2 steel.

Elements	C	Mn	P	S	Si	Cr	V	Mo
wt.%	1.4–1.6	0.1–0.6	0.03	0.03	0.1–0.6	11–13	0.5–1.1	0.7–1.2

**Table 2:** Physical and mechanical properties of AISI D2 steel.

Property	Value
Density (kg/m³)	7700
Melting Point (°C)	1421
Elastic Modulus (GPa)	190
Tensile Strength (MPa)	2000
Compressive Strength (MPa)	1650
Poisson’s Ratio	0.28
Hardness (HRC)	60
Thermal Conductivity (W/m·°C)	20
Specific Heat (J/kg·°C)	460
Electrical Resistivity (Ω·m)	5.79 × 10^−7^

**Fig 1:**
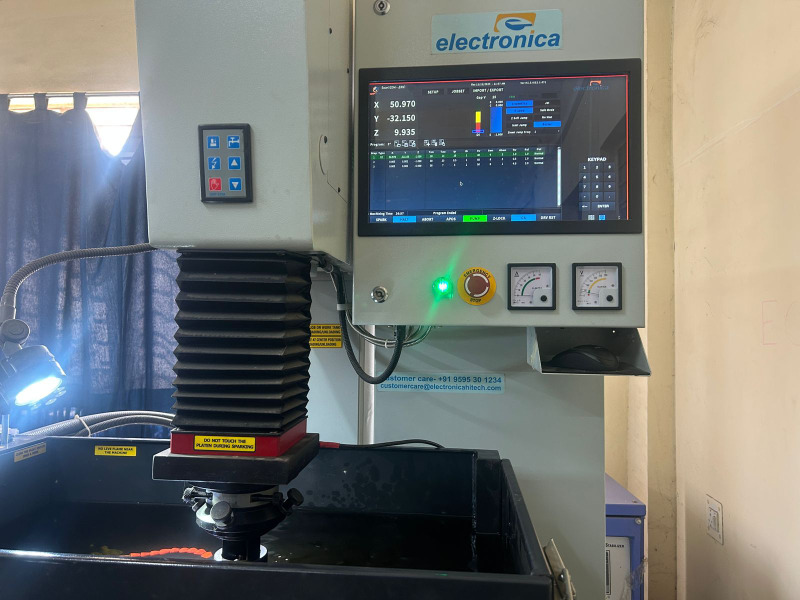
Experimental setup of EDM.

**Fig 2:**
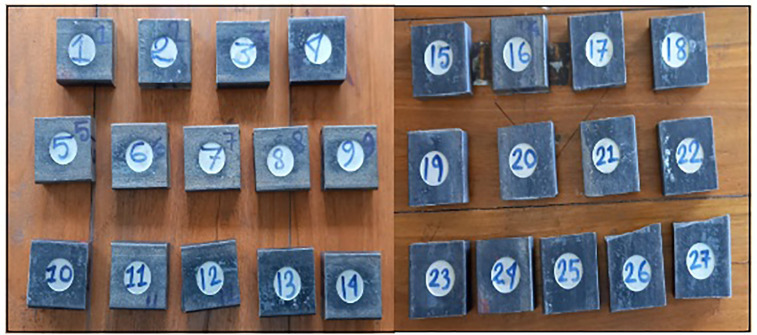
Machined workpiece.

All experiments were carried out on an ELTECH D300 ZNC (ELECTRONICA) die-sinking EDM machine shows in Fig 1. The workpieces were held firmly using a magnetic chuck. A copper electrode of 25 mm diameter was employed, chosen for its high electrical and thermal conductivity. Commercially available EDM oil was used as the dielectric fluid owing to its excellent dielectric strength, high flash point, and good thermal stability. There is no electrode rotation. The dielectric was circulated into the inter-electrode gap using a pumping system to ensure effective flushing of debris. Fig 2 shows the machined specimens.

### Process parameters and experimental design

Peak current (Ip), pulse-on time (Ton), and duty cycle (c) were selected as the process parameters because these parameters are directly governing the discharge energy and spark characteristics in EDM, which predominantly control material removal rate and surface integrity. Peak current influences the intensity of the discharge and thus crater size and MRR, while pulse-on time controls the duration of energy input per spark, affecting both MRR and surface roughness. Duty cycle regulates the ratio of spark-on to spark-off time, influencing debris flushing efficiency and thermal stability of the machining zone. These three parameters are consistently reported in the literature as the most influential and practically controllable electrical variables in EDM of tool steels. Moreover, limiting the study to three parameters allows effective modelling of main, interaction, and quadratic effects using the Box–Behnken design with a reasonable number of experiments, ensuring statistical robustness while maintaining experimental feasibility. This also reduced number of experiments, avoidance of extreme factor combinations. Table 3 shows the three EDM process parameters and their three levels.

**Table 3:** EDM process parameters and their levels.

Parameter	Level 1	Level 2	Level 3
Peak Current, Ip (A)	10	20	30
Pulse-on Time, Ton (µs)	10	30	50
Duty Cycle, c (%)	5	10	15

**Fig 3:**
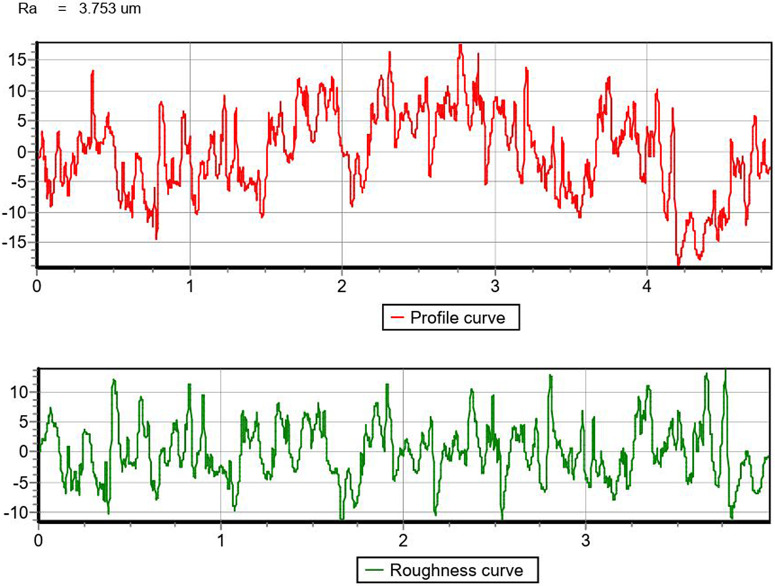
Surface roughness profiles (experiment no 11).

The experimental results are presented in Table 4. For each experimental trial, a blind hole of 1 mm depth was machined on the specimen surface using an electrode of corresponding diameter. The performance measures considered were Material Removal Rate (MRR) and Surface Roughness (SR). The MRR was calculated using Equation (1), based on the difference in specimen weight before and after machining, along with the recorded machining time. Surface roughness was measured using an INSIZE MN-ISR-C300-E surface roughness tester. For each specimen, three readings were taken at different locations, and the average value was reported to minimize measurement uncertainty. The surface roughness profile for Experiment No. 11 is shown in Fig 3.

**Table 4:** Experimental results of EDM.

Experiment Sl No.	Peak Current (Ip)	Pulse on Time (Ton)	Duty Cycle(c)	w1(weight before machining)	w2 (weight after machining)	Machining time (min)	MRR (g/min)	SR (µm)	SR2 (µm)	SR3 (µm)	AVG SR (µm)
1	10	10	5	413.25	410.7	10.02	0.254	2.881	2.843	2.952	2.892
2	10	10	10	407.7	405.1	9	0.289	3.093	3.127	3.172	3.131
3	10	10	15	417.95	415.6	7.59	0.309	3.3	3.073	3.349	3.241
4	10	30	5	415.25	411.25	13.55	0.295	4.959	4.85	4.759	4.856
5	10	30	10	421.5	417.9	11.17	0.322	5.167	4.734	4.701	4.867
6	10	30	15	421.07	418.3	8.01	0.346	5.321	5.511	5.5052	5.445
7	10	50	5	415.55	412.27	10.41	0.315	5.498	6.629	6.063	6.063
8	10	50	10	409.84	407	8.51	0.333	6.103	5.233	6.157	5.831
9	10	50	15	419.46	416.5	8.25	0.359	5.961	5.233	5.929	5.708
10	20	10	5	420.9	416.6	14.26	0.301	3.587	3.605	3.404	3.532
11	20	10	10	413.08	408.54	14.1	0.322	3.47	3.753	3.347	3.523
12	20	10	15	416.45	412.36	12.24	0.334	3.895	3.027	3.974	3.632
13	20	30	5	423.21	419.3	12.01	0.325	5.822	5.369	6.268	5.82
14	20	30	10	419.69	415.82	11.42	0.339	5.969	4.791	5.822	5.527
15	20	30	15	416.28	412.47	11.01	0.346	6.566	4.544	5.008	5.373
16	20	50	5	421.57	418.21	9.48	0.354	6.881	6.899	7.123	6.968
17	20	50	10	417.85	414.49	9.01	0.372	7.22	6.899	6.887	7.002
18	20	50	15	415.73	412.53	8.45	0.379	7.464	7.181	7.354	7.333
19	30	10	5	424.1	419.1	15.51	0.322	3.227	4.603	3.275	3.702
20	30	10	10	418.38	413.6	14.32	0.334	3.687	3.031	3.533	3.417
21	30	10	15	414.7	409.92	13.57	0.352	3.85	3.888	4.067	3.935
22	30	30	5	416.02	412.41	10.56	0.341	6.075	6.174	5.552	5.934
23	30	30	10	418.88	415.23	10.23	0.356	4.992	6.484	6.092	5.856
24	30	30	15	422.96	419.52	9.48	0.362	6.145	5.721	5.851	5.906
25	30	50	5	418.82	414.85	11.05	0.359	6.325	7.305	7.206	6.945
26	30	50	10	419.91	416.11	10.32	0.368	6.047	6.545	6.909	6.5
27	30	50	15	411.93	408.35	9.39	0.381	5.749	6.765	5.694	6.069

The MRR was determined using the following relation:


$$\begin{array}{c} \text{MRR }(\text{g}/\text{min})=\frac{\left(w_{\text{1}}-w_{\text{2}}\right)}{t} \end{array}$$ (1)

where

$w_{\text{1}}$ = weight of the specimen before machining (g),

$w_{\text{2}}$ = weight of the specimen after machining (g),

t = machining time (min).

### Response surface methodology

Response Surface Methodology (RSM) is a powerful statistical and mathematical tool used to model and analyze response parameters influenced by multiple process variables [21–24]. It is widely applied in engineering optimization problems to establish empirical relationships between input parameters and corresponding output responses. RSM typically employs a second-order polynomial model, which relates the response variable to the independent process variables, as shown in Equation (2).


$$\begin{array}{c} \hat{\text{Y}}=\beta_{\text{0}}+\sum \beta_{i}X_{i}+\sum \beta_{ii}\overset{\text{2}}{\underset{i}{X}}+\sum \beta_{iii}X_{i}X_{j}+\varepsilon \end{array}$$ (2)

Where Ŷ represent the response, X_i_ is the independent effect of the variable, X_j_ correlation between the variable, β is the model coefficient, k is the number of variable and ε is the error associated with experiment.

Several experimental design strategies, such as Box–Behnken Design (BBD), Central Composite Design (CCD), and Latin Hypercube Design (LHD), are commonly used in RSM. In the present study, the selected process parameters are peak current (Ip), pulse-on time (Ton), and duty cycle (c). The second-order polynomial model used in this work is expressed in Equation (3).


$$\begin{array}{c} \hat{\text{Y}}=\beta_{\text{0}}+\beta_{\text{1}}I_{p}+\beta_{\text{2}}T_{on}+\beta_{\text{3}}c+\beta_{\text{1}\text{1}}\overset{\text{2}}{\underset{p}{I}}+\beta_{\text{2}\text{2}}\overset{\text{2}}{\underset{on}{T}}+\beta_{\text{3}\text{3}}c^{\text{2}}+\beta_{\text{1}\text{2}}I_{p}T_{on}+\beta_{\text{2}\text{3}}T_{on}c+\beta_{\text{3}\text{1}}cI_{p} \end{array}$$ (3)

This equation establishes the relationship between the EDM process parameters and the responses under investigation, namely Material Removal Rate (MRR) and Surface Roughness (SR).

### Non-dominated sorting genetic algorithm II (NSGA-II)

The Non-Dominated Sorting Genetic Algorithm II (NSGA-II) is one of the most widely used multi-objective optimization techniques due to its ability to generate diverse and well-distributed Pareto-optimal solutions [25]. Unlike single-objective optimization methods,

NSGA-II simultaneously optimizes multiple conflicting objectives by employing evolutionary principles such as selection, crossover, and mutation.

**Fig 4:**
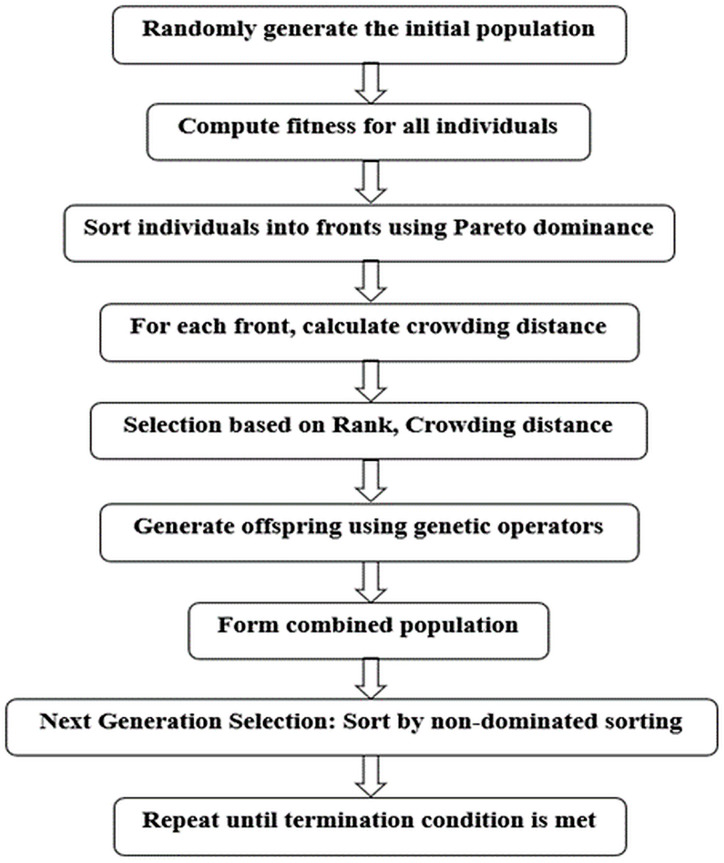
Flow diagram of NSGA-II algorithm [23].

The algorithm begins with a randomly generated population and ranks individuals based on non-dominated sorting. Selection is performed considering both the rank and the crowding distance, which ensures diversity among solutions. The parent and offspring populations are combined, and the best solutions are passed on to the next generation. This iterative process continues until convergence is achieved, yielding a Pareto front of optimal solutions. The overall procedure of NSGA-II is illustrated in Fig 4.

### Technique for order preference by similarity to ideal solution (TOPSIS)

Single-objective optimization methods yield a unique optimal solution. In contrast, multi-objective optimization problems generally involve mutually conflicting objectives, resulting in a set of optimal solutions rather than a single optimum. These solutions, referred to as Pareto-optimal or non-dominated solutions, are such that no solution is superior to another when all objectives are considered simultaneously. Therefore, an additional decision-making approach is required to identify the most suitable solution for practical implementation.

The Technique for Order of Preference by Similarity to Ideal Solution (TOPSIS) [26] is a widely used multi-criteria decision-making (MCDM) method for selecting the most desirable alternative from a set of non-dominated solutions. The fundamental principle of TOPSIS is that the optimal solution should be closest to the positive ideal solution and farthest from the negative ideal solution. In the present study, TOPSIS is employed to rank the Pareto-optimal solutions obtained from NSGA-II and to identify the most preferred machining parameter combination.

The procedure adopted for TOPSIS implementation is summarized as follows:

**Step 1: Construction and normalization of the decision matrix**

The TOPSIS is one of the simple, comprehensive and rational ranking method. In this process, the units of all objectives is eliminated and normalized value is taken. The Pareto solutions are denoted by S and the elemental value of S_ij_ is the j^th^ objective of the i^th^ solution. The elemental value of S_ij_ is normalized according to the following equations:


$$\begin{array}{c} {\text{S}\sim }_{ij}=\frac{S_{ij}}{\sqrt{\sum_{i}^{m}\overset{\text{2}}{\underset{ij}{S}}}}\text{ i}=\text{1},\text{2},\ldots ..,\text{m};\text{ j}=\text{1},\text{2} \end{array}$$ (4)

**Step 2: Calculation of weighted normalized matrix**

Calculate the normalized weighted value by multiplication of normalized rating (Sij) and weight (W_j_).


$$\begin{array}{c} {\hat{\text{S}}}_{\text{ij}}={\tilde{\text{S}}}_{ij}\times Wj\text{ i}=\text{1},\text{2},\ldots ..,\text{m};\text{ j}=\text{1},\text{2} \end{array}$$ (5)

The weight Wj is the value of j^th^ attribute and it must obey following condition:


$$\begin{array}{c} \sum_{j=\text{1}}^{\text{2}}Wj=\text{1} \end{array}$$ (6)

**Step 3: Determination of ideal best and ideal worst solutions**

Find out the ideal best (S^+^) and ideal worst (S^-^) according to the following condition:


S+=(min (S^11,S^21,S^31,……,S^m1),  max(S^12,S^22,S^32,……S^m2)) (7)

$$\begin{array}{c} {\text{S}}^{-}=(\text{max }\left(\hat{\text{S}}\text{11},\hat{\text{S}}\text{21},\hat{\text{S}}\text{31},\ldots \ldots ,\hat{\text{S}}\text{m1}\right),\text{ max}\left(\hat{\text{S}}\text{12},\hat{\text{S}}\text{22},\hat{\text{S}}\text{32},\ldots \ldots \hat{\text{S}}\text{m2}\right)) \end{array}$$ (8)

**Step 4: Distance measures from ideal solutions**

Calculate the distance measure h^+^ and h^-^ for each alternative from ideal best (S^+^) and ideal worst (S^-^) using following equations:


$$\begin{array}{c} \overset{+}{\underset{i}{h}}=\sqrt{\sum_{j=\text{1}}^{\text{2}}{\left({\hat{\text{S}}}_{\text{ij}}-S^{+}\right)}^{\text{2}}\text{ i}=\text{1},\text{2},\ldots \ldots ,\text{m}} \end{array}$$ (9)

$$\begin{array}{c} \overset{-}{\underset{i}{h}}=\sqrt{\sum_{j=\text{1}}^{\text{2}}{\left({\hat{\text{S}}}_{\text{ij}}-S^{-}\right)}^{\text{2}}}\text{ i}=\text{1},\text{2},\ldots \ldots ,\text{m} \end{array}$$ (10)

**Step 5: Calculation of relative closeness**

Find out the relative closeness (Hi) of each Pareto solution using following equation:


$$\begin{array}{c} H_{i}=\frac{\overset{-}{\underset{i}{h}}}{\overset{+}{\underset{i}{h}}+\;\overset{-}{\underset{i}{h}}} \end{array}$$ (11)

**Step 6: Selection of the optimal solution**

Select best Pareto solution whose relative closeness (Hi) is nearest to 1.

## Results and discussion

### Effect of process parameters on MRR

The ANOVA results and standard regression coefficient for MRR are presented in Tables 5 and 6 respectively at a 95% confidence level and the results showing that the regression model for MRR is highly significant (F = 67.84, p < 0.001). Both the linear and interaction effects of current, pulse-on time, and duty cycle are highlighted. Current, pulse-on time, and duty cycle are highly significant where each “p” value has less than 0.001. Current, pulse on time and duty cycle all have small but positive coefficient 0.0072, 0.002028 and 0.0082 indicating that responses tend to increases with increasing these variables. Among these parameters, pulse-on time (Ton) was identified as the most influential factor it’s contributing 39.44% (F value 247.5) to MRR, followed by current 30.26% (F value 192.83) and duty cycle 22.14% (F value 189.89). The P-value indicates the significance of the coefficients of each input parameter. If P-value is lower or equal to 0.05, it is normally regarded as statistically significant. In this work the model “p” value was found to be less than 0.001, indicating statistical significance and confirming that the considered parameters have a substantial impact on the response.

**Table 5:** Analysis of variance (ANOVA) results for material removal rate (MRR).

Source	DF	Coefficient	Adj SS	Adj MS	T-Value	F-Value	P-Value	Percent Contribution
Model	9		0.022257	0.002473		67.84	0	
Linear	3		0.021012	0.007004		192.13	0	
Current(Ip)	1	0.0072	0.006923	0.006923	3.93	189.89	0	0.3026
Pulse on(Ton)	1	0.002028	0.009023	0.009023	7.58	247.5	0	0.3944
Duty Cycle(c)	1	0.0082	0.005067	0.005067	−0.1	138.99	0	0.2214
Square	3		0.000438	0.000146		4	0.025	
current(Ip)*current(Ip)	1	−0.000082	0.0004	0.0004	−3.23	10.98	0.004	0.0174
Pulse on(Ton)*Pulse on(Ton)	1	−0.000004	0.000014	0.000014	−4.42	0.37	0.551	0.00061
Duty Cycle(c)*Duty Cycle(c)	1	−0.00008	0.000024	0.000024	0.93	0.66	0.428	0.00104
2-Way Interaction	3		0.000807	0.000269		7.38	0.002	
current(Ip)*Pulse on(Ton)	1	−0.000023	0.000252	0.000252	0.908	6.91	0.018	0.0110
current(Ip)*Duty Cycle(c)	1	−0.000128	0.000494	0.000494	0.244	13.55	0.002	0.02159
Pulse on(Ton)*Duty Cycle(c)	1	−0.000022	0.000061	0.000061	0.154	1.67	0.214	0.00266
Error	17		0.00062	0.000036				
Total	26		0.022877					

**Table 6:** Standard regression coefficient of MRR.

Term	Coef	SE Coef	T-Value	P-Value	VIF
Constant	0.1471	0.0166	8.86	0	
Ip (A)	0.0072	0.00109	6.62	0	58.38
Ton (µs)	0.002028	0.00045	4.51	0	40
c (%)	0.0082	0.00217	3.77	0.002	58.37
Ip (A)*Ip (A)	−0.000082	0.000025	−3.31	0.004	49
Ton (µs)*Ton (µs)	−0.000004	0.000006	−0.61	0.551	28
c (%)*c (%)	−0.00008	0.000099	−0.81	0.428	49
Ip (A)*Ton (µs)	−0.000023	0.000009	−2.63	0.018	10.38
Ip (A)*c (%)	−0.000128	0.000035	−3.68	0.002	13
Ton (µs)*c (%)	−0.000022	0.000017	−1.29	0.214	10.37

**Fig 5:**
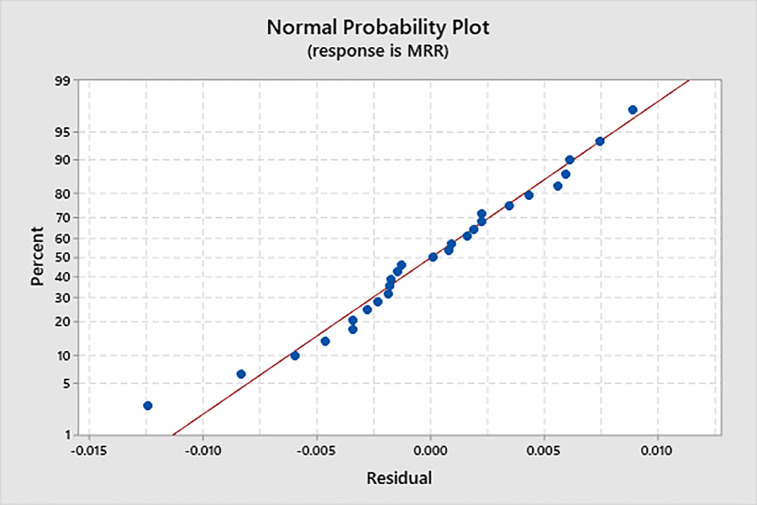
Normal probability plot of residuals for MRR.

**Fig 6:**
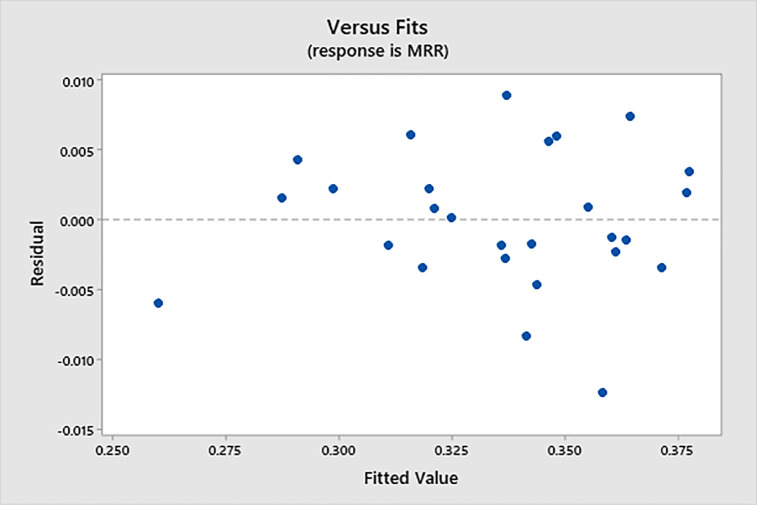
Versus fits plot of fitted values for MRR.

The normal probability plot of residuals for MRR (Fig 5) shows that the residuals closely follow a straight line and the experimental values (blue colour dots) of MRR obtained were sliding along the diagonal line designated the residuals were shared normally for the generated regression model. Fig 6 shows the match type of predicted MRR and experimental values of MRR. Nearly all the entire experimental values (blue colour dots) were distributed to the horizontal line that designates the residuals was a perfect match and it indicating constant variance for the generated regression model. No data points were excluded from the analysis.

**Fig 7:**
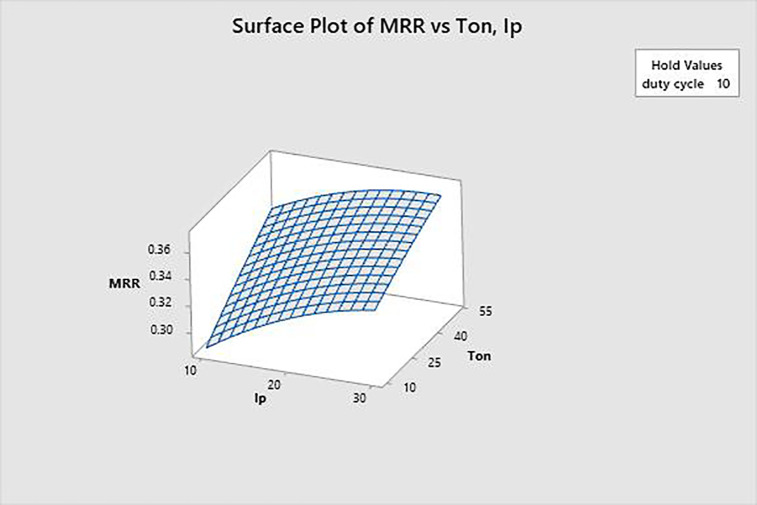
Surface plot MRR Vs current and pulse on time.

**Fig 8:**
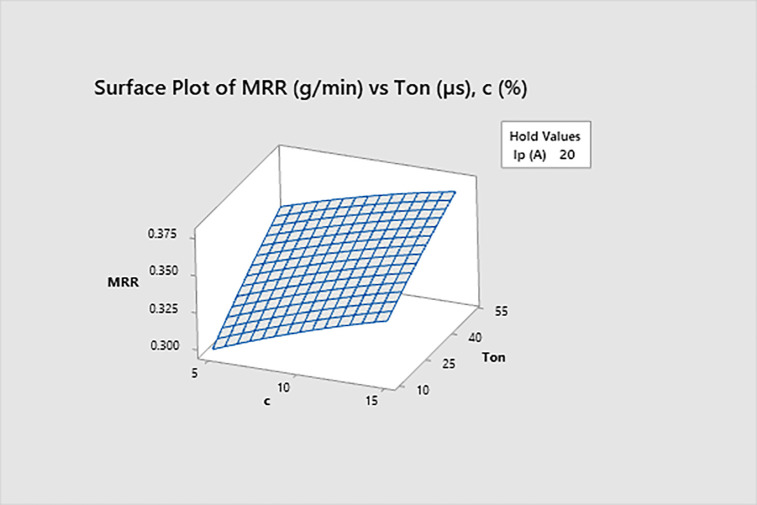
Surface plot MRR Vs duty cycle and pulse on time.

The 3D surface plots generated from the predictive model (Figs 7 and 8) provide additional insights into the effect of process variables. Fig 7 reveals that MRR increases with higher values of both current and pulse-on time when the duty cycle is fixed at 10%. The surface plot shows a remarkable upward curvature which means combined effect of both variables longer pulse on time and higher current leads to a significantly higher MRR. At elevated pulse-on time, the material removal rate is maximized due to higher discharge energy. Pulse-on-time is the duration of pulse allowed to flow per cycle. As Ton increases more energy applied resulting high temperature created at sparking zone due to this larger and deeper crater formed and more material removed from the workpiece [26]. Fig 8 illustrates the influence of duty cycle and pulse-on time while keeping current constant at 20Amp A. It indicating that MRR values lower at shorter pulse duration and lower duty cycle. The overall upward trend shows that MRR increases with increase in duty cycle and pulse on time. The duty cycle exhibits only a minor effect on MRR compared to pulse-on time, further validating the ANOVA results.

### Effect of process parameters on SR

The ANOVA results for surface roughness (SR) are summarized in Table 7. Current and pulse-on-time have positive value of coefficient 0.2123 and 0.1692 respectively it indicating that responses SR increases with increasing these two variables. “P” value has also less than 0.001 for above two process parameters it shows that these two variables are highly significant. Duty cycle coefficient value has negative (−0.011) and also the p value has 0.957 which is greater than 0.05 it indicating that duty cycle has no significant effect on SR. It is evident that pulse-on time is the dominant factor, contributing 86% (F value 464.87), followed by current 4.44% (F value 24.01). With longer pulse-on time, sparks of higher energy are generated, leading to deeper and wider craters on the workpiece surface, which consequently increases SR. Table 8 also shows the standard regression coefficient.

**Table 7:** Analysis of variance (ANOVA) results for surface roughness (SR).

Source	DF	Coef	Adj SS	Adj MS	F-Value	P-Value	Percent contribution
Model	9		47.0151	5.2239	58.16	0.000	
Linear	3		43.9081	14.6360	162.96	0.000	
Current (Ip)	1	0.2123	2.1563	2.1563	24.01	0.000	0.04442
Pulse on(Ton)	1	0.1692	41.7515	41.7515	464.87	0.000	0.86011
Duty Cycle(c)	1	−0.011	0.0003	0.0003	0.00	0.957	0.000006
Square	3		2.7751	0.9250	10.30	0.000	
Current (Ip)*Current(Ip)	1	−0.00396	0.9393	0.9393	10.46	0.005	0.01935
Pulse on(Ton)*Pulse on(Ton)	1	−0.001353	1.7583	1.7583	19.58	0.000	0.03622
Duty Cycle(c)*Duty Cycle(c)	1	0.00455	0.0775	0.0775	0.86	0.366	0.00159
2-Way Interaction	3		0.3320	0.1107	1.23	0.329	
Current (Ip)*Pulse on (Ton)	1	0.000051	0.0012	0.0012	0.01	0.908	0.000002
Current (Ip)*Duty Cycle(c)	1	−0.00209	0.1310	0.1310	1.46	0.244	0.00269
Pulse on (Ton)*Duty Cycle(c)	1	−0.00129	0.1997	0.1997	2.22	0.154	0.00411
Error	17		1.5268	0.0898			
Total	26		48.5420				

**Table 8:** Standard regression coefficient of MRR.

Term	Coef	SE Coef	T-Value	P-Value	VIF
Constant	−0.392	0.824	−0.48	0.64	
Ip (A)	0.2123	0.054	3.93	0.001	58.38
Ton (µs)	0.1692	0.0223	7.58	0	40
c (%)	−0.011	0.108	−0.1	0.918	58.37
Ip (A)*Ip (A)	−0.00396	0.00122	−3.23	0.005	49
Ton (µs)*Ton (µs)	−0.001353	0.000306	−4.42	0	28
c (%)*c (%)	0.00455	0.00489	0.93	0.366	49
Ip (A)*Ton (µs)	0.000051	0.000433	0.12	0.908	10.38
Ip (A)*c (%)	−0.00209	0.00173	−1.21	0.244	13
Ton (µs)*c (%)	−0.00129	0.000865	−1.49	0.154	10.37

**Fig 9:**
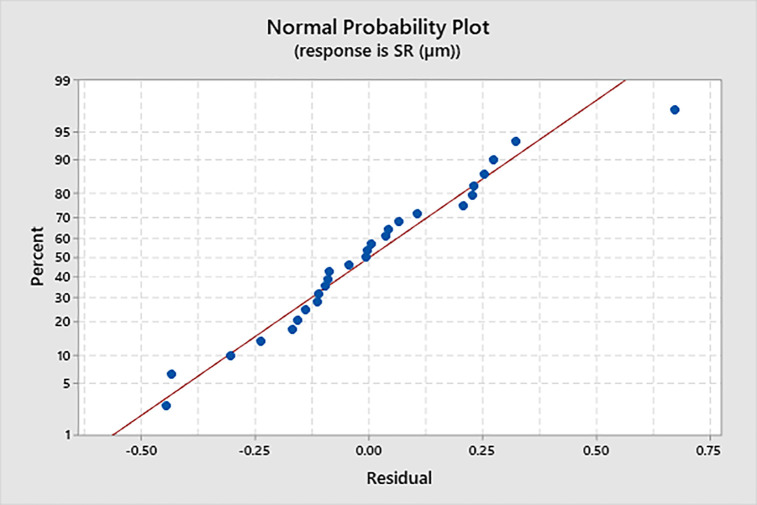
Normal probability plot of residuals for SR.

**Fig 10:**
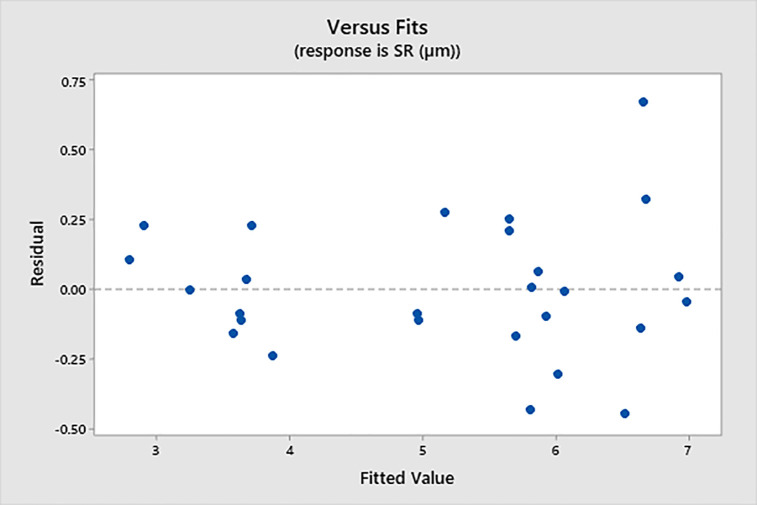
Versus fits plot of fitted values for SR.

The normal probability plot of residuals for SR (Fig 9) shows that the residuals closely follow a straight line and the experimental values (blue colour dots) of SR obtained were sliding along the diagonal line designated the residuals were shared normally for the generated regression model. Fig 10 shows the match type of predicted SR and experimental values of SR. Nearly all the entire experimental values were distributed to the horizontal line (dotted line) that designates the residuals was a perfect match and it indicating constant variance for the generated regression model. No influential points were excluded from the analysis.

**Fig 11:**
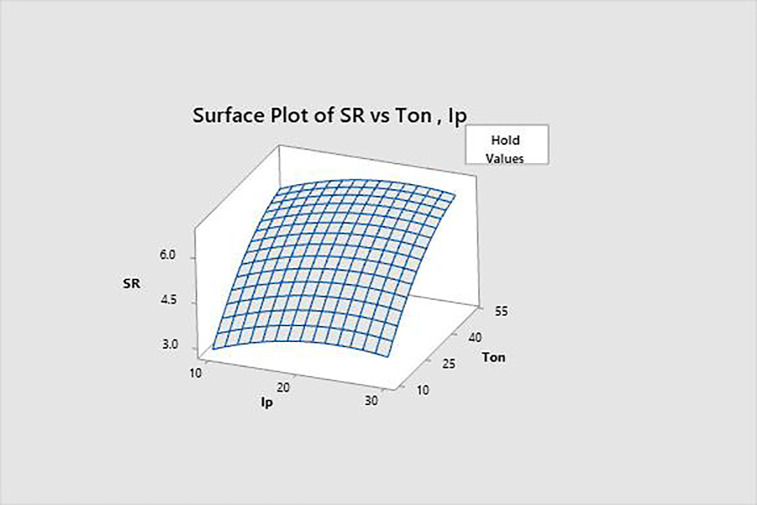
Surface plot SR Vs current and pulse on time.

**Fig 12:**
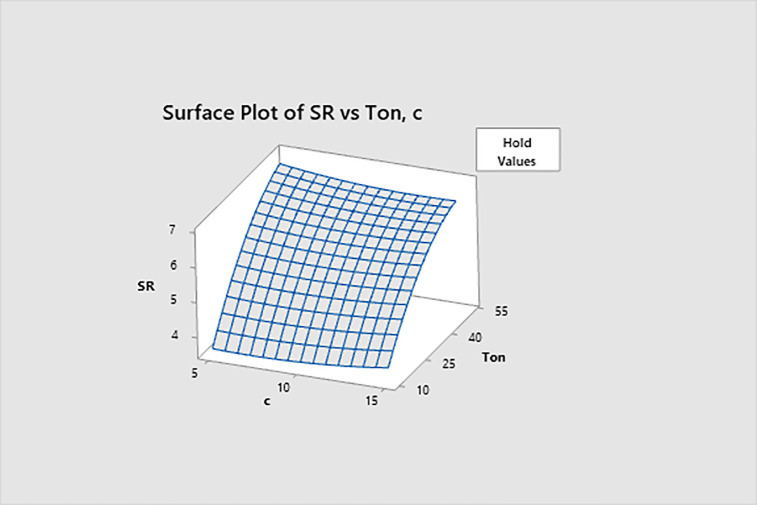
Surface plot SR Vs duty cycle and pulse on time.

The surface plots (Figs 11 and 12) further explain the parametric effects. Fig 11 shows that SR initially increases slightly and then decreases with an increase in current, whereas a consistent rise in SR is observed with an increase in pulse-on time. As current increases plasma channel temperature and pressure increases as number of ion and electron per unit area increases at sparking zone resulting larger volume of material removed from the workpiece. Hence surface roughness increases or rough machined surface formed as each discharged forms larger and deeper crater when current and pulse-on-time increases [27–30]. Fig 12 indicates that the duty cycle has minimal influence on SR when compared to the pulse-on-time and current.

Table 9 shows the model summary for both MRR and SR. R^2^ values for both models are close to 100% which are 97.29% (MRR) and 96.85% (SR). It shows the excellent model fit. Further adjusted R^2^ and predicted R^2^ for both model was found more than 90% it also indicating the developed regression models provide an excellent fit with experimental data and selected process parameters significantly influence the MRR and SR.

**Table 9:** Model summary.

Response	Standard deviation	R-sq	R-sq.(adj)	R-sq.(pred)
MRR	0.0060379	97.29%	95.86%	93.51%
SR	0.299690	96.85%	95.19%	91.75%

Based on the response surface methodology (RSM), quadratic regression models were developed for both MRR and SR. These models account for the individual as well as interactive effects of process parameters:


$$\begin{array}{cc} MRR=\; & \text{0}.\text{1}\text{4}\text{7}\text{1}+\text{0}.\text{0}\text{0}\text{7}\text{2}\text{0}I_{p}+\text{0}.\text{0}\text{0}\text{2}\text{0}\text{2}\text{8}T_{on}+\text{0}.\text{0}\text{0}\text{8}\text{2}\text{0}c-\text{0}.\text{0}\text{0}\text{0}\text{0}\text{8}\text{2}\overset{\text{2}}{\underset{p}{I}}-\text{0}.\text{0}\text{0}\text{0}\text{0}\text{0}\text{0}\text{4}\overset{\text{2}}{\underset{on}{T}}-\text{0}.\text{0}\text{0}\text{0}\text{0}\text{8}\text{0}c^{\text{2}}\\ & \;\;\;-\text{0}.\text{0}\text{0}\text{0}\text{0}\text{2}\text{3}I_{p}T_{on}-\text{0}.\text{0}\text{0}\text{0}\text{2}\text{2}T_{on}c-\text{0}.\text{0}\text{0}\text{0}\text{1}\text{2}\text{8}cI_{p} \end{array}$$ (12)

$$\begin{array}{cc} SR= & -\text{0}.\text{3}\text{9}\text{2}+\text{0}.\text{2}\text{1}\text{2}\text{3}I_{p}+\text{0}.\text{1}\text{6}\text{9}\text{2}T_{on}-\text{0}.\text{0}\text{1}\text{1}c-\text{0}.\text{0}\text{0}\text{3}\text{9}\text{6}\overset{\text{2}}{\underset{p}{I}}-\text{0}.\text{0}.\text{0}\text{0}\text{1}\text{3}\text{5}\text{3}\overset{\text{2}}{\underset{on}{T}}+\text{0}.\text{0}\text{0}\text{4}\text{5}\text{5}c^{\text{2}}\\ & +\text{0}.\text{0}\text{0}\text{0}\text{0}\text{5}\text{1}I_{p}T_{on}-\text{0}.\text{0}\text{0}\text{1}\text{2}\text{9}\text{0}T_{on}c-\text{0}.\text{0}\text{0}\text{2}\text{0}\text{9}cI_{p} \end{array}$$ (13)

These predictive models are statistically adequate and provide a strong foundation for subsequent multi-objective optimization using the hybrid RSM–NSGA-II approach.

### Multi objective optimization using NSGA-II

In the present study, Material Removal Rate (MRR) and Surface Roughness (SR) are considered as the important performance indicators of the Electrical Discharge Machining (EDM) process. These responses are inherently conflicting in nature. The ANOVA results indicate that pulse-on time (Ton) is the most influential parameter affecting both MRR and SR, followed by peak current (Ip). An increase in pulse-on time and peak current leads to higher discharge energy, which enhances the material removal rate due to the formation of larger and deeper craters. However, this also results in a deterioration of surface quality, as the enlarged crater size, re-solidified layer, and possible micro-cracks contribute to increased surface roughness. Conversely, lower discharge energy produces finer craters, improving surface finish at the expense of reduced MRR. This clearly establishes the trade-off between machining efficiency and surface integrity. To address this multi-objective conflict, the quadratic RSM models developed for MRR and SR (Eqs. 12–13) were utilized as fitness functions in the Non-dominated Sorting Genetic Algorithm II (NSGA-II). The optimization problem was formulated to simultaneously maximize MRR and minimize SR within the specified process parameter constraints.

The optimization problem was defined as follows:

- Objective 1: Maximize MRR (Eq. 12)
- Objective 2: Minimize SR (Eq. 13)

Subject to the constraints on input process parameters:

Peak current I_p_ (Amp): 10 ≤ I_p_ ≤ 30
Pulse on time T_on_ (µs): 10 ≤ T_on_ ≤ 50
Duty Cycle (c): 5 ≤ c ≤ 15

**Fig 13:**
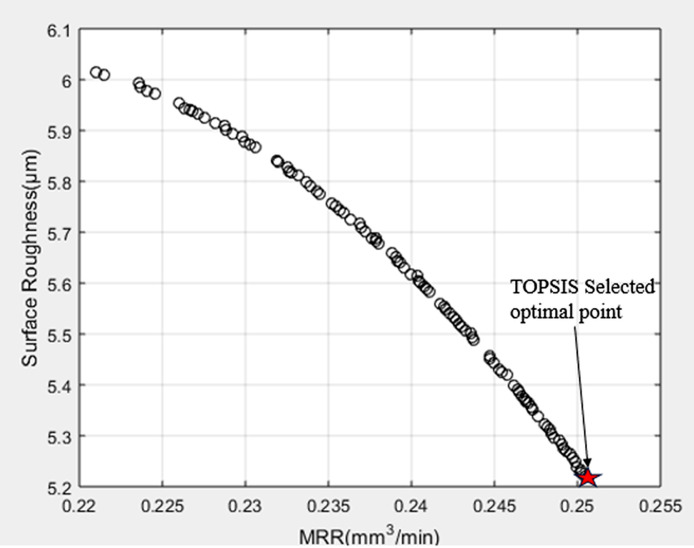
Pareto optimal solutions and TOPSIS selected optimal point.

NSGA-II generated a set of Pareto-optimal solutions representing the trade-off frontier between MRR and SR. Table 10 presents the Pareto-optimal solutions and their corresponding objective values. The Pareto front (Fig 13) clearly demonstrates that improvement in one objective leads to deterioration in the other, confirming the conflicting nature of the responses. To select the most suitable operating condition, the Technique for Order Preference by Similarity to Ideal Solution (TOPSIS) was applied, assigning equal importance to both objectives.

**Table 10:** Pareto optimal solutions.

Current (Ip)	Pulse on (Ton)	Duty Cycle(c)	MRR_2_	SR
10.01	49.95	14.96	6.01	0.22
10.03	30.70	14.94	5.22	0.25
10.02	30.80	14.96	5.23	0.25
10.03	31.53	14.96	5.28	0.25
10.02	49.66	14.95	6.01	0.22
10.04	30.90	14.92	5.23	0.25
10.03	31.21	14.85	5.25	0.25
10.02	40.34	14.96	5.74	0.24
10.03	31.52	14.79	5.27	0.25
10.02	40.49	14.97	5.75	0.24
10.02	39.87	14.98	5.73	0.24
10.02	34.26	14.96	5.44	0.24
10.03	34.42	14.96	5.45	0.24
10.02	31.84	14.98	5.30	0.25
10.03	48.93	14.79	5.99	0.22
10.02	35.56	14.94	5.51	0.24
10.03	33.28	14.95	5.39	0.25
10.03	31.43	14.95	5.27	0.25
10.02	33.10	14.93	5.37	0.25
10.02	35.61	14.97	5.52	0.24
10.01	47.57	14.96	5.97	0.22
10.02	44.02	14.98	5.88	0.23
10.02	46.40	14.98	5.94	0.23
10.02	42.33	14.96	5.82	0.23
10.03	31.76	14.84	5.28	0.25
10.02	35.05	14.96	5.49	0.24
10.02	40.18	14.96	5.74	0.24
10.03	31.29	14.87	5.26	0.25
10.02	37.06	14.93	5.59	0.24
10.02	32.99	14.96	5.37	0.25
10.03	32.52	14.96	5.34	0.25
10.02	43.70	14.96	5.87	0.23
10.03	32.72	14.96	5.35	0.25
10.02	46.19	14.95	5.94	0.23
10.02	45.23	14.97	5.91	0.23
10.01	43.94	14.95	5.87	0.23
10.01	49.95	14.96	6.01	0.22
10.02	39.34	14.96	5.70	0.24
10.02	32.26	14.95	5.32	0.25
10.02	38.90	14.96	5.68	0.24
10.02	35.75	14.96	5.53	0.24
10.03	45.07	14.92	5.91	0.23
10.02	41.27	14.95	5.78	0.23
10.07	37.35	14.96	5.61	0.24
10.02	30.95	14.96	5.24	0.25
10.03	32.93	14.95	5.36	0.25
10.02	44.61	14.95	5.89	0.23
10.02	35.14	14.95	5.49	0.24
10.03	35.87	14.96	5.53	0.24
10.02	39.49	14.95	5.71	0.24
10.02	38.45	14.91	5.66	0.24
10.03	35.42	14.94	5.51	0.24
10.02	34.02	14.91	5.43	0.25
10.02	34.05	14.96	5.43	0.25
10.03	38.06	14.96	5.64	0.24
10.02	32.82	14.94	5.36	0.25
10.02	48.24	14.96	5.99	0.22
10.02	39.05	14.97	5.69	0.24
10.03	42.78	14.96	5.84	0.23
10.03	39.06	14.91	5.69	0.24
10.02	37.77	14.96	5.63	0.24
10.03	35.38	14.87	5.50	0.24
10.02	44.76	14.98	5.90	0.23
10.02	38.31	14.89	5.65	0.24
10.02	41.53	14.94	5.79	0.23
10.02	41.09	14.97	5.77	0.23
10.02	36.83	14.96	5.58	0.24
10.03	39.79	14.87	5.72	0.24
10.02	36.92	14.96	5.59	0.24
10.01	47.96	14.96	5.98	0.22
10.03	36.01	14.96	5.54	0.24
10.03	33.55	14.91	5.40	0.25
10.02	36.19	14.95	5.55	0.24
10.02	37.24	14.96	5.60	0.24
10.03	42.04	14.96	5.81	0.23
10.03	33.36	14.95	5.39	0.25
10.03	32.20	14.92	5.32	0.25
10.02	42.85	14.96	5.84	0.23
10.02	40.65	14.96	5.76	0.24
10.04	46.73	14.96	5.95	0.23
10.02	42.25	14.96	5.82	0.23
10.03	32.09	14.93	5.31	0.25
10.02	46.22	14.97	5.94	0.23
10.04	44.38	14.91	5.89	0.23
10.04	42.46	14.96	5.83	0.23
10.03	36.29	14.94	5.55	0.24
10.03	37.53	14.96	5.62	0.24
10.02	31.95	14.96	5.30	0.25
10.02	33.17	14.94	5.38	0.25
10.02	38.03	14.93	5.64	0.24
10.02	31.85	14.86	5.29	0.25
10.02	36.33	14.97	5.56	0.24
10.03	33.88	14.90	5.42	0.25
10.04	41.69	14.96	5.80	0.23
10.02	38.81	14.96	5.68	0.24
10.03	37.18	14.96	5.60	0.24
10.02	45.87	14.98	5.93	0.23
10.02	45.69	14.95	5.93	0.23
10.03	34.48	14.95	5.46	0.24

The optimal solution obtained through TOPSIS is highlighted in Fig 13, and corresponding results are presented in Table 11. The analysis reveals that at Ip = 10.03 A, Ton = 30.70 µs, and duty cycle = 14.94%, a balanced trade-off is achieved, yielding an MRR of 0.25 g/min and an SR of 5.22 µm.

**Table 11:** TOPSIS selected optimized results.

Responses		Input Factors		
Surface Roughness (µm)	MRR (g/min)	Peak current I_p_ (Amp)	Pulse on time T_on_(µs)	Duty Cycle (c)
5.22	0.250	10.033	30.70044	14.94291

This indicates that a combination of lower peak current, moderate pulse-on time, and higher duty cycle provides a desirable compromise between machining efficiency and surface quality. The moderate discharge energy under these conditions promotes controlled crater formation, resulting in acceptable material removal while maintaining improved surface integrity.

The corresponding optimal decision parameters and responses are summarized in Table 11. The results indicate that at a peak current of 10.03 A, a pulse-on time of 30.70 µs, and a duty cycle of 14.94%, a desirable trade-off is achieved, yielding an MRR of 0.25 g/min and an SR of 5.22 µm. The results shows that combination of lower level of peak current, medium level of pulse on time and higher level of duty cycle give optimized MRR and surface roughness.

### Confirmation of optimal solutions

To verify the accuracy of the optimized experimental parameter settings, confirmation tests were conducted. The confirmation test was executed as per obtained process parameters listed in Table 11, and the experimental values were compared against the predicted values in Table 12. The observed error percentages for Roughness (µm) and MRR (mm^3^/min) were 3.9% and 4.8%, respectively. These low error rates confirm that the RSM-NSGA-II-TOPSIS approach effectively optimizes the EDM of AISI D2 Steel.

**Table 12:** Confirmation test result.

Predicted Surface Roughness (µm)	Experimental Surface Roughness (µm)	Percentage of Error	Predicted MRR (g/min)	Experimental MRR (g/min)	Percentage of Error
5.22	5.424	3.9	0.25	0.262	4.8

## Conclusion

In this study, the hybrid RSM–NSGA-II–TOPSIS framework for systematically used to address the conflicting objectives inherent in EDM of AISI D2 steel. The integrated methodology provides a structured decision-making approach which statistically validated models with evolutionary optimization and multi-criteria ranking, and give practical guidance for selecting machining conditions that balance productivity and surface integrity.

Despite its effectiveness, the present investigation is subject to certain limitations. The analysis was restricted to three electrical process parameters and a shallow blind-hole depth, which may limit direct extrapolation of the findings to deeper cavities or complex industrial geometries. In addition, electrode wear rate and thermal damage characteristics were not explicitly considered, and experiments were conducted under controlled laboratory conditions with a limited number of repetitions.

Future work may focus on incorporating additional performance indicators such as electrode wear, tool life, and subsurface integrity, as well as extending the study to greater machining depths and different electrode and dielectric materials. The proposed framework can also be integrated with real-time monitoring, adaptive control strategies, or machine-learning-based surrogate models to further enhance its applicability to advanced and intelligent manufacturing systems.
